# The association of actigraphic sleep measures and physical activity with excess weight and adiposity in kindergarteners

**DOI:** 10.1038/s41598-021-82101-x

**Published:** 2021-01-27

**Authors:** Justyna Wyszyńska, Piotr Matłosz, Agnieszka Szybisty, Katarzyna Dereń, Artur Mazur, Jarosław Herbert

**Affiliations:** 1 Institute of Health Sciences, Medical College, Rzeszow University, Rzeszow, Poland; 2 Centre for Innovative Research in Medical and Natural Sciences, University of Rzeszow, Rzeszow, Poland; 3 Institute of Physical Culture Sciences, Medical College, Rzeszow University, Rzeszow, Poland; 4 Institute of Medical Sciences, Medical College, Rzeszow University, Rzeszow, Poland

**Keywords:** Risk factors, Obesity, Paediatric research

## Abstract

Insufficient sleep duration and physical activity (PA) are known risk factors for overweight and obesity in children; however, there are no studies on comprehensive associations of objectively-measured sleep parameters and PA with excess weight and excess adiposity in kindergarteners. Therefore, the aim of this study was to determine the associations between objectively measured sleep parameters and PA with excess weight and excess adiposity, defined as BMI ≥ 85th percentile and body fat percentage (BFP) ≥ 85th percentile, respectively. Sleep parameters and PA were measured in 676 subjects aged 5–6 years using accelerometers for 7 days, worn at the participant’s hip. Bioelectrical impedance analysis was used to estimate BFP. In the total sample, lower sleep duration, sleep efficiency, vigorous PA and the number of steps per day were associated with excess weight. However, excess adiposity was associated with lower sleep duration, total PA, vigorous PA, moderate-to-vigorous physical activity (MVPA) and the number of steps per day. Logistic regression by the stepwise progressive method showed that the strongest predictor of excess adiposity in boys and girls was vigorous PA, while the strongest predictor of excess weight in boys was sleep efficiency. A holistic approach to health targeting all of these factors synergistically is needed to optimize the effectiveness of obesity prevention and treatment interventions.

## Introduction

The increasing prevalence of pediatric overweight and obesity has become a well-recognized public health problem in Europe as well as in other developed countries^1^. Worldwide, over 41 million children under 5 years of age are estimated to be overweight or obese^2^. Evidence shows that early childhood obesity tracks into adulthood^3^. This has important public health consequences because excess weight in childhood is closely related to non-communicable diseases in adulthood, including diabetes, hypertension, cardiovascular diseases and specific types of cancer^4^. Therefore, identifying factors associated with childhood overweight and obesity is an urgent public health need.

Industrialization, mechanized transport, urbanization and changes in lifestyle over the last decades have caused an increase in the prevalence of obesity^5^. One of these changes concerns sleep habits^6,7^ and the quality of sleep affected by an increased use of electronic devices before sleep^8^. Inadequate sleep in early childhood is related to multiple consequences, including cognitive disruptions, attention difficulties, poor school performance and mood disturbances^9,10^. Changes in sleep patterns have also been suggested as a potential contributor to the obesity epidemic in both adults and children^11^. The extant research focusing primarily on sleep duration does not provide sufficient information on the role of sleep in childhood obesity. Sleeping less than nine hours a day has been reported to be a risk factor for excess weight in children aged from 2 to 18 years^12^. A meta‐analysis indicated that children and adolescents from 5 to 18 years old sleeping for a short duration (i.e., < 9 h per day for school-aged children and < 8 h per day for teenagers) had twice the risk (OR = 2.15) of being overweight or obese, compared with their peers sleeping for a longer duration (i.e., > 11 h per day for school-aged children and > 10 h per day for teenagers)^13^. However, sleep duration is not the only sleep parameter that might contribute to weight gain. Other parameters like sleep efficiency, number of awakenings or wake after sleep onset (WASO) might also contribute to body mass^14^.

A recent study, including 1.6 million pupils aged 11–17 years, reported that in 2016, 19% reach the World Health Organization (WHO) recommended level of at least 60 min of moderate-to-vigorous physical activity (MVPA) per day, with more boys (22.4%) being active than girls (15.3%)^15^. In most European countries, less than 50% of children and adolescents meet these recommendations^16^. Insufficient physical activity (PA) has been linked to overweight and obesity^17^. The combined prevalence of overweight/obesity in European children below the age of 10 ranges from more than 40% in southern Europe to less than 10% in northern Europe. Overall, the prevalence of overweight was higher in girls (21.1%) as compared with boys (18.6%)^1^. However, to the best of our knowledge, the literature contains no reports on detailed associations between sleep parameters and PA among young children aged five to six years old in the context of obesity. Understanding the association of PA and sleep parameters with body mass and adiposity in kindergarteners is important for future strategies to prevent obesity.

Current evidence so far has often been limited to studies using self-reported sleep analysis that include mainly sleep duration and body mass index (BMI) to identify obesity in infants, children and adolescents^18^. Moreover, studies using objective indicators have indicated inconsistent results^19,20^. To the best of our knowledge, there are no reports with a comprehensive analysis of objectively measured sleep parameters and PA with excess weight and excess adiposity among a large sample of kindergarteners. Therefore, we explored the associations between objectively measured sleep parameters and PA among 5 to 6-year-old subjects with excess weight and excess adiposity.

## Methods

### Participants

This cross-sectional study was carried out from October to November 2018–2019 in Rzeszów, Poland. The details of the study design and a partial analysis have been reported elsewhere^21^. In brief, the study was conducted at 22 kindergartens in Rzeszów, Poland. Written informed consent was obtained from parents or legal guardians and the participating children prior to participation in the study. The study was approved by the Bioethics Committee at the Medical Faculty of the University of Rzeszów, decision no. 2018/01/05 on 11 January 2018, and it was conducted in accordance with the ethical standards laid down in the relevant version of the Declaration of Helsinki. The study included 676 healthy participants aged 5 to 6 years.

### Accelerometer measurements

The ActiGraph GT3X-BT tri-axial accelerometer (ActiGraph, Pensacola, Florida, USA) was used to objectively measure sleep parameters and PA. Data were analyzed using the ActiLife 6.13 data analysis software. Accelerometers have been used extensively to measure PA and are considered to be a valid measure of free-living PA in preschool children^22^. Moreover, actigraphy is a valid, effective and cost-efficient alternative to polysomnography^23^. The accelerometer was worn at the participant’s right hip. Participants were instructed to wear the monitor continually for 7 consecutive days and nights, removing it only for water-related activities^24^.

The sleep-period time (from sleep onset to the end of sleep, including all sleep epochs and wakefulness after onset) was detected and separated from 24-h activity using a Tudor-Locke algorithm^25^. Similarly to our study, this algorithm was designed for accelerometer data integrated into 60 s epochs, collected 24 h per day for 7 consecutive days using a right-hip-worn ActiGraph GT3X + in young children.

#### Physical activity

After the exclusion of sleep-period time, non-wear time was defined as a period of at least 60 consecutive minutes of zero counts allowing for 2 min of non-zero interruptions^26^. In this study, the epoch duration was set to 5 s, which has been shown to be more accurate for the assessment of the spontaneous and intermittent activities of preschool children^27^*.* A minimum wear time of ≥ 500 min/day was considered a valid day for the analysis, and at least 4 days were used as the criteria for a valid seven-day period of accumulated data (including at least three valid weekdays and one valid weekend day)^28^. For each child, the total volume of PA (total counts/wear time in minutes, cpm), time (min/day) spent in light PA, moderate PA, vigorous PA, MVPA and the mean daily step count were calculated. The cut-off points from Evenson et al. were used to determine the time spent during light (101–2295 cpm), moderate (2296–4011 cpm), vigorous (≥ 4012 cpm) and MVPA (≥ 2296 cpm from all valid days)^29^. The majority of children (n = 741; 77.8%) provided 7 valid days of actigraphic data; 170 (17.8%) and 42 (4.4%) children had 6 and 5 valid days of actigraphic data, respectively. Average monitoring included 6.7 days of recordings.

#### Sleep parameters

For analysis of sleep parameters, the ‘Sadeh’ sleep algorithm was used^30^. In this study, the following four sleep outcomes were examined: sleep duration, sleep efficiency, wake after sleep onset (WASO) and number of awakenings. Sleep duration (min) refers to the number of 60 s epochs in a sleep episode scored as ‘sleep’, excluding any time scored as ‘wake’. Sleep efficiency (percentage) was calculated as the amount of time the participants were asleep over the amount of time they were in bed. WASO (min) refers to periods of wakefulness occurring after sleep onset. Number of awakenings is defined as the frequency of awakenings during sleep-period time^31^.

### Anthropometric measurements

Body height was measured with a stadiometer (Tanita HR-200, Tokyo, Japan) to the nearest 0.1 cm under standard conditions. Body height was measured barefoot, in an upright position with upper back, buttocks and heels against a stadiometer, when the head was held in the Frankfort plane. Body mass was measured with a body composition analyzer (BC-420 MA, Tanita, Tokyo, Japan). The participants were measured in light clothing and wearing no shoes. Individual BMI was calculated as body mass (kg) divided by height (m) squared. Polish BMI charts specific for age, sex and body height were used to determine individual BMI percentiles^32^. Children were classified into two groups: (1) ‘normal weight’ (BMI percentile < 85th percentile) and (2) ‘excess weight’ (overweight/obesity) (≥ 85th percentile)^33^.

### Body composition

Body fat percentage was obtained using a bioelectrical impedance analysis (BIA) method by foot-to-foot body composition analyzer (BC-420 MA, Tanita). The participants’ parents had been instructed about the necessity of at least an 8 h overnight fast before the examination^34^.

The foot-to-foot BIA device showed a moderate to strong correlation with dual-energy X-ray absorptiometry (DEXA) for BFP, and high specificity for overweight and obese classifications in children aged from 5 to 11 years (Intraclass correlation coefficients, ICC = 0.788)^35^. A systematic review assessing the validity, responsiveness, reliability and measurement error of BIA methods in estimating BFP in children and adolescents suggests that BIA is a practical method to estimate BFP in the pediatric population^36^. For the purpose of this study, the participants were classified as: (1) ‘no excess adiposity’ (BFP percentile < 85th percentile) and (2) ‘excess adiposity’ (≥ 85th percentile). For this purpose, BFP charts by age and sex were used^37^.

### Socio-demographic data

Socio-demographic characteristics (children’s sex and date of birth) were self-reported by the parents/caregivers.

### Statistical analysis

Statistical analysis was performed using SPSS 20 software (IBM, North Harbour, UK). The data were presented as the mean ± standard deviation (SD), median, interquartile range (IQR) and percentage (%) for continuous and categorical variables, respectively. Normality was tested by the Kolmogorov–Smirnov test. None of the analyzed variables showed compliance with a normal distribution, therefore non-parametric tests were applied. The chi-squared and the Mann–Whitney U tests were used to compare two groups of participants divided based on BMI and BFP percentiles. The analyses were also performed in subgroups based on sex. Logistic regression analysis by the stepwise progressive method was used to identify factors associated with excess weight and excess adiposity. Logistic regression was performed separately for boys and girls. The following continuous variables were included in the models as predictors of excess weight and excess adiposity: age, total PA, light PA, moderate PA, vigorous PA, MVPA, steps per day, sleep duration, sleep efficiency, WASO and number of sleep awakenings^38,39^. The level of statistical significance was set at p < 0.05.

## Results

The general characteristic of the study population, according to sex, is shown in Table 1. Excess adiposity was more prevalent in boys than girls (55% vs. 43.3%, respectively) (p < 0.001). The mean of total PA, light PA, moderate PA, MVPA and steps per day were higher in boys compared to girls (606 vs. 582 cpm, 470.4 vs. 464.2 min/day, 42.2 vs. 35.1 min/day, 51.0 vs. 44.2 min/day and 9375 vs. 8999, respectively) (p < 0.05).

**Table 1:** General characteristics of the study population.

Variable	Boys *(n* = 331)	Girls *(n* = *345)*	All subjects *(n* = 676)	*p*
Age (years)^a^	5.6 ± 0.5 (6; 1)	5.6 ± 0.5 (6; 1)	5.6 ± 0.5 (6; 1)	0.742
Body height (cm)^a^	117.9 ± 6.1 (118.0; 8.5)	116.5 ± 5.9 (116.6; 8.1)	117.2 ± 6.1 (117.2; 8.5)	**0.006**
Body mass (kg)^a^	22.1 ± 4.1 (21.5; 4.8)	21.3 ± 3.6 (20.7; 4.6)	21.7 ± 3.9 (21.1; 4.9)	**0.008**
BMI (kg/m^2^)^a^	15.8 ± 1.8 (15.5; 1.9)	15.6 ± 1.7 (15.3; 2.1)	15.7 ± 1.8 (15.3; 2.0)	0.147
**Classification based BMI percentiles**^b^				
Normal weight	293 (88.5)	307 (89.0)	600 (88.8)	0.848
Excess weight	38 (11.5)	38 (11.0)	76 (11.2)	
BFP (%)^a^	20.3 ± 4.7 (19.3; 4.4)	20.4 ± 4.8 (19.8; 5.9)	20.4 ± 4.7 (19.5; 5.1)	0.291
**Classification based BFP percentiles**^b^				
No excess adiposity	149 (45.0)	234 (67.8)	383 (56.7)	** < 0.001**
Excess adiposity	182 (55.0)	111 (32.2)	293 (43.3)	
Total PA (cpm)^a^	606 ± 147 (610; 160)	582 ± 131 (574; 166)	594 ± 139 (596; 165)	**0.002**
Light PA (min/day)^a^	470.4 ± 86.5 (479.1; 78.3)	464.2 ± 66.6 (470.3; 62.0)	467.2 ± 77.0 (473.8; 70.9)	**0.012**
Moderate PA (min/day)^a^	42.2 ± 19.1 (39.8; 21.3)	35.1 ± 14.6 (33.6; 19.6)	38.6 ± 17.3 (36.7; 20.9)	** < 0.001**
Vigorous PA (min/day)^a^	9.7 ± 9.0 (7.7; 7.9)	9.7 ± 8.6 (7.3;7.9)	9.7 ± 8.8 (7.5; 7.9)	0.838
MVPA (min/day)^a^	51.0 ± 23.9 (48.3; 29.0)	44.2 ± 20.5 (40.7; 25.3)	47.5 ± 22.5 (44.5; 27.5)	** < 0.001**
Steps/day^a^	9375 ± 2548 (9167; 2974)	8999 ± 2071 (8731; 2757)	9183 ± 2323 (8997; 2999)	**0.034**
Sleep duration (min)^a^	570.1 ± 62.5 (576.7; 80.4)	577.5 ± 69.7 (584.1; 78.2)	573.9 ± 66.3 (579;6; 76;3)	0.077
Sleep efficiency (%)^a^	97.2 ± 1.7 (97.6; 1.7)	97.4 ± 1.3 (97.6; 1.6)	97.3 ± 1.5 (97.6; 1.7)	0.280
WASO (min)^a^	14.6 ± 6.7 (13.4; 9.0)	14.0 ± 6.0 (13.4; 8.5)	14.3 ± 6.3 (13.4; 8.5)	0.392
Number of awakenings^a^	5 ± 2.0 (5.0; 2.7)	5 ± 2.0 (4.7; 2.6)	5 ± 2.0 (4.9; 2.7)	0.122

Data are expressed as: ^a^mean ± SD (median; IQR); ^b^n (%); *p* value represents the differences between boys and girls.

*BFP* body fat percentage, *BMI* body mass index, *MVPA* moderate to vigorous physical activity, *PA* physical activity, *WASO* wake after sleep onset; significant associations are highlighted in bold. Activity levels were determined based on triaxial 24 h-accelerometry vector magnitude, defining 101–2295 cpm as light PA, 2296–4011 cpm as moderate PA, ≥ 4012 cpm as vigorous PA and ≥ 2296 cpm as MVPA^29^. Excess weight was defined as BMI ≥ 85th percentile; normal weight was defined as BMI < 85th percentile^33^. Excess adiposity was defined as BFP ≥ 85th percentile; no excess adiposity was defined as BMI < 85th percentile^37^.

Table 2 shows the distribution of PA and sleep parameters among participants classified according to BMI and BFP percentiles. Subjects with excess weight compared to peers with normal weight had lower vigorous PA (8.3 vs. 9.9 min/day), steps per day (8680 vs. 9246), sleep efficiency (96.9 vs. 97.4%) and sleep duration (546.9 vs. 577.3 min). When considering children with excess adiposity, it was found that their values of total PA, vigorous PA, MVPA, steps per day and sleep duration were lower, compared to those with no excess adiposity (579 vs. 606 cpm, 8.0 vs. 11.0 min/day, 45.3 vs. 49.3 min/day, 8829 vs. 9454 and 565.5 min vs. 580.3 min, respectively).

**Table 2:** Physical activity and sleep parameters among children with different body weight and adiposity levels based on the 85th percentile.

Variable		Total PA (cpm)	Light PA (min/day)	Moderate PA (min/day)	Vigorous PA (min/day)	MVPA (min/day)	Steps/ day	Sleep efficiency (%)	Sleep duration (min)	WASO (min)	Number of awakenings
**Classification based BMI percentiles**											
Normal weight (293 boys; 307 girls)	Mean	596	467.3	38.9	9.9	48.0	9246	97.4	577.3	14.2	5.0
	SD	141	77.2	17.1	8.9	22.1	2330	1.4	64.3	6.2	2.0
	Median	598	473.3	37.0	7.6	45.1	9031	97.7	582.9	13.3	5.0
	IQR	164	71.3	20.9	8.0	28.1	2865	1.6	75.5	8.0	3.0
Excess weight (38 boys; 38 girls)	Mean	579	466.5	36.0	8.3	44.0	8680	96.9	546.9	15.4	6.0
	SD	130	75.7	19.1	7.9	25.1	2219	2.1	75.8	6.9	3.0
	Median	582	475.7	34.3	6.3	41.3	8324	97.1	551.1	16.1	5.0
	IQR	178	71.2	20.8	6.9	27.3	3029	2.5	94.4	11.2	4.0
*p*		0.223	0.715	0.095	**0.049**	0.072	**0.026**	**0.014**	** < 0.001**	0.133	0.162
**Classification based BFP percentiles**											
No excess adiposity (149 boys; 234 girls)	Mean	606	464.5	39.2	11.0	49.3	9454	97.4	580.3	14.1	5.0
	SD	147	83.1	17.3	10.0	22.8	2370	1.4	65.1	6.3	2.0
	Median	606	471.6	37.4	8.3	46.0	9151	97.6	586.0	13.4	5.0
	IQR	168	71.9	22.4	9.0	29.7	2878	1.6	69.3	8.2	3.0
Excess adiposity (182 boys; 111 girls)	Mean	579	470.9	37.8	8.0	45.3	8829	97.2	565.5	14.5	5.0
	SD	127	68.1	17.3	6.6	21.9	2214	1.6	67.1	6.4	2.0
	Median	580	475.8	36.4	6.6	43.1	8746	97.5	574.6	13.7	5.0
	IQR	154	66.9	18.4	7.0	23.9	2773	1.8	82.6	8.9	3.0
*p*		**0.015**	0.764	0.281	** < 0.001**	**0.032**	** < 0.001**	0.133	**0.005**	0.433	0.162

*BFP* body fat percentage, *BMI* body mass index, *MVPA* moderate to vigorous physical activity, *PA* physical activity, *WASO* wake after sleep onset; significant associations are highlighted in bold. Activity levels were determined based on triaxial 24 h-accelerometry vector magnitude, defining 101–2295 cpm as light PA, 2296–4011 cpm as moderate PA, ≥ 4012 cpm as vigorous PA and ≥ 2296 cpm as MVPA^29^. Excess weight was defined as BMI ≥ 85th percentile; normal weight was defined as BMI < 85th percentile^33^. Excess adiposity was defined as BFP ≥ 85th percentile; no excess adiposity was defined as BMI < 85th percentile^37^.

Details of the distribution of PA and sleep parameters among boys and girls, classified according to BMI and BFP percentiles, respectively, are presented in Supplementary tables S1–S2. Boys with excess adiposity had lower levels of all PA parameters except for light PA, whereas girls with excess adiposity accumulated a lower number of steps per day and minutes per day of vigorous PA compared to those with no excess adiposity. In groups stratified by BMI, boys with excess weight had lower vigorous PA, steps per day, sleep duration and higher WASO and number of awakenings, while in girls with excess weight only lower sleep duration was found compared to girls with normal weight.

Table 3 shows a logistic regression analysis by the stepwise progressive method to identify factors associated with excess weight and excess adiposity. In boys, excess weight was associated with lower sleep efficiency (OR = 0.77, p = 0.003). It was also found that excess adiposity was associated with lower vigorous PA both in girls and boys (p = 0.021 and p < 0.001, respectively).

**Table 3:** Variables influencing excess weight and excess adiposity—logistic regression by the stepwise progressive method.

Variables		B	SE	Wald	df	*p*	OR (95% CI)
**Excess weight**							
Girls	Constant	− 2.09	0.17	147.60	1.00	** < 0.001**	0.12
Boys	Sleep efficiency (%)	− 0.25	0.09	8.88	1.00	**0.003**	0.77 (0.66–0.92)
**Excess adiposity**							
Girls	Vigorous PA (min/day)	− 0.04	0.02	5.34	1.00	**0.021**	0.96 (0.93–0.99)
Boys	Vigorous PA (min/day)	− 0.06	0.02	13.07	1.00	** < 0.001**	0.94 (0.91–0.97)

*df* degrees of freedom, *PA* physical activity, *OR (95% CI)* odds ratio with a 95% confidence interval, *SE* standard error; significant associations are highlighted in bold. Results were derived from logistic regression by the stepwise progressive method of analysis with factors potentially related to excess weight and excess adiposity. B-coefficient can be interpreted as a predictor of the impact on the dependent variable. The model included age, total PA, light PA, moderate PA, vigorous PA, MVPA, steps per day, sleep duration, sleep efficiency, WASO and number of sleep awakenings. The last step of the regression model with the variables that best explained the analyzed relationships was presented. Activity levels were determined based on triaxial 24 h-accelerometry vector magnitude, defining 101–2295 cpm as light PA, 2296–4011 cpm as moderate PA, ≥ 4012 cpm as vigorous PA and ≥ 2296 cpm as MVPA^29^.

## Discussion

To our knowledge, this is the first study to examine comprehensive associations between objectively measured PA, sleep parameters and classification, according to BMI and BFP percentiles, in a relatively large sample of kindergarteners. The present results showed that in the total sample of children aged from 5 to 6 years, lower vigorous PA, number of steps per day, sleep duration and sleep efficiency were associated with excess weight. Excess adiposity was also associated with lower vigorous PA, steps per day and sleep duration, but also with lower total PA and MVPA. Logistic regression by the stepwise progressive method showed that the strongest predictor of excess adiposity in boys and girls was vigorous PA, while the strongest predictor of excess weight in boys was sleep efficiency. Additionally, previously reported findings from this cohort indicated differences in content of BFP, fat-free mass (FFM) and total body water (TBW) between boys reaching and not reaching the WHO recommended level of MVPA. Accumulating at least 60 min of MVPA per day was associated with a twofold lower risk of excess BFP (≥ 95th percentile) in boys but not in girls^21^.

The association between sleep duration, PA and weight status was analyzed by Ji et al. among Chinese children aged from three to five years. Sleep duration and PA were measured by the Misfit Shine 2 fitness wristband. The results of their study indicated higher prevalence of excess weight in boys compared to girls. Moreover, children with excess weight were more likely to sleep less than 8 hours^40^. Previous studies also suggested that a lack of sufficient sleep and sleeping late among children might contribute to excess weight^10,41^. Rosi et al. found no differences among BMI groups for all the lifestyle factors in Italian adolescents. Moreover, no relationship was found between body weight and diet, PA, sleep duration adequacy (“low if less than 8 h per night, adequate if between 8 and 11 h per night or high if more than 11 h per night”), sleep quality and school achievement. All data in their study were self-reported^42^. In our study, more apparent associations between sleep parameters and PA with weight and adiposity were observed in boys. Boys with excess weight had lower sleep duration, sleep efficiency, vigorous PA and number of steps per day, as well as higher WASO and number of awakenings than those with normal weight. In girls with excess weight, sleep duration was lower by 30 min a day on average, compared to girls with normal weight. However, excess adiposity was associated in boys with lower level of PA, measured by total PA, moderate PA, vigorous PA, MVPA and number of steps per day, by approximately 40 cpm, 5 min/day, 4 min/day, 8 min/day and 1000 steps daily, respectively. In girls, excess adiposity was associated with lower vigorous PA by 2.35 min/day and lower number of steps per day by almost 500 steps. Nonetheless, no associations were found in WASO, number of awakenings and all PA parameters except vigorous PA in the total sample of children stratified by BMI. In children stratified by BFP null results were also observed in light and moderate PA, and parameters related with quality of sleep (WASO, number of awakenings and sleep efficiency). In boys, divided based on BMI percentiles, null results were found in all PA parameters except vigorous PA, while in girls null results were found in all analyzed PA and sleep parameters except sleep duration. However, in boys, divided based on BFP percentiles, no associations were found in light PA and all analyzed sleep parameters, and in girls additionally in total PA, moderate PA and MVPA.

The mechanism of association between sleep duration and adiposity is not clear; however, existing evidence has suggested that it may be related to hormones and PA^43^. Insufficient sleep may lead to endocrine alteration. The sympathetic nervous system can be activated by changes in the levels of hormones such as leptin, ghrelin, insulin and cortisol, causing an increase in appetite, food intake and as a result an energy excess^44^. Insufficient sleep may also contribute to specific behavior, such as a low level of PA and high levels of sedentary behavior and food intake^45,46^. In pre-schoolers, total sleep time is inversely associated with sedentary time^47^. Average sleep duration has been decreasing worldwide for decades, potentially contributing to the increase in the prevalence of obesity ^48^. A potential explanation for the association of sleep with excess weight that should be considered is sleep disordered breathing (e.g., sleep apnea), one of the consequences of childhood obesity, which occurs in up to 60% of obese children in the USA. In turn, sleep disordered breathing results in decreased sleep duration and quality (e.g. poor sleep efficiency and more WASO) which are associated with increases in body weight and adiposity^49,50^. Since insufficient sleep is a modifiable risk factor, it should be considered in the prevention and treatment of childhood obesity.

Besides sleep duration, other sleep indicators, such as sleep quality, efficiency and daytime sleepiness, have been individually examined with obesity, albeit less frequently^51^. In children, poor sleep quality (e.g. self-reported sleep disturbances or difficulty in initiating sleep) has also been associated with excess weight in several cross-sectional studies^52^. However, objective indicators of sleep quality, such as efficiency, are relatively rarely used. Our study indicated that lower sleep efficiency was associated with excess weight in the total sample of kindergarteners. Moreover, in boys with excess weight, besides lower sleep duration and efficiency, longer WASO and higher number of awakenings were also observed. Slightly different relationships have been observed when adiposity was assessed. Boys with excess adiposity had lower values of total PA, moderate PA, vigorous PA, MVPA and steps per day. These differences can be explained by the fact that BMI is an indirect measure of adiposity and does not distinguish between fat and fat-free mass, in contrast with more direct approaches such as BIA. Moreover, it has been recognized that individuals with a similar BMI can vary considerably in their adiposity^53^. On the other hand, evidence indicated that the BIA device can overestimate BFP^54^. In the study by Tyrrell et al. BIA-BFP overestimated DEXA-BFP by a mean of 2.53%^55^.

Self-reported sleep quality was inversely associated with children’s body weight^52^, whereas studies using device-based parameters indicated inconsistent results^19,20^. Opposite results to ours were obtained by Xiu et al., who found no difference in sleep parameters among children aged from two to six years of age^56^. However, the results of a systematic review showed that children with obesity accumulated less than 60 min of MVPA per day, which was less than children with normal weight^57^. MVPA in the pediatric population is generally below the recommended 60 min/day. It is necessary to make efforts to encourage children and adolescents to reach the recommended levels of MVPA for prevention and treatment of childhood obesity.

Our study has several strengths and limitations. A limitation of this study is the cross-sectional design, which meant that causal pathways underlying the observed relationships could not be assessed. Moreover, we cannot be certain that confounding factors, such as nutrition or genetic factors, have not influenced our observations. Although measures of energy intake would have enriched our ability to draw conclusions from the results, the lack of those measures and the failure to obtain strong associations between sleep parameters and PA with body weight and adiposity do not negate the relevance of this study to public health. Another limitation of this study was the use of BIA instead of DEXA, which is considered to be the gold standard in estimation of BFP. Despite the good reliability of BIA methods in estimating BFP in children and adolescents, the validity and measurement error are not satisfactory^36^. Moreover, Tyrrell et al. indicated that BIA-BFP overestimated DEXA-BFP by a mean of 2.53% (the limits of agreement are 4.29% and 9.36%)^55^. However, considering feasibility and the safety of participants, the authors decided to use BIA instead of a DEXA assessment. In addition, in our study we did not use national sex-specific centile curves for BFP. Due to the lack of Polish centile curves for BFP, we used centile curves by McCarthy et al. that were established for a pediatric population from Manchester in the UK^37^. The next limitation of our study was a single measurement of body height. Hip worn accelerometers for assessment of sleep quality metrics can be recognized as another limitation of our study. However, accelerometers placed at the hip seems to be superior for sleep timing and quantity metrics compared to wrist worn accelerometers^58^. Another limitation of this study is the fact that participants’ tonsillectomy procedures were not recorded, as these are especially important in children around age 5, because greatly enlarged tonsils may obstruct the airway and cause sleep apnea^59^.

The study, however, benefits from a number of strengths, including a relatively large sample size and a focus on sleep and PA in kindergarteners. Moreover, measurements of sleep and PA using actigraphy provided objective evidence of the associations of sleep characteristics and PA with body weight and adiposity beyond mere sleep duration. The 5 s epoch used in this study appears to capture a greater amount of data in preschool children. In addition, we examined the above associations using not only BMI classification, but also BFP.

Future research should include a greater emphasis on longitudinal studies to examine how objectively measured sleep parameters and adiposity vary by age within an individual over time, taking into account the impact of sleep disordered breathing and possible confounding factors.

## Conclusions

Sleep efficiency and vigorous PA are associated with excess weight and excess adiposity in kindergarteners. A holistic approach to health, targeting all of these behaviors synergistically, is needed to optimize the effectiveness of obesity prevention and treatment interventions. Assessment of obesity using objective methods is recommended.

## Supplementary Information

Supplementary Tables.

## Data Availability

All data generated or analyzed during this study are included in this published article.
