# Supplementary material for: The association of actigraphic sleep measures and physical activity with excess weight and adiposity in kindergarteners
Source: Sci Rep. 2021 Jan 27;11:2298. doi: 10.1038/s41598-021-82101-x (PMC7840732; doi:10.1038/s41598-021-82101-x)
Supplement: Supplementary file 1 — Supplementary Tables. [file 41598_2021_82101_MOESM1_ESM.docx]

**The association of actigraphic sleep measures and physical activity with excess weight and adiposity in kindergarteners**

Justyna Wyszyńska, Piotr Matłosz, Agnieszka Szybisty, Katarzyna Dereń , Artur Mazur and Jarosław Herbert

Supplementary table S1. Physical activity and sleep parameters in boys with different body weight and adiposity levels based on the 85th percentile.

| **Variable** | | | **Total PA (cpm)** | **Light PA (min/day)** | **Moderate PA (min/day)** | **Vigorous PA (min/day)** | **MVPA (min/day)** | **Steps/ day** | **Sleep efficiency (%)** | **Sleep duration (min)** | **WASO (min)** | **Number of awakenings** |
| --- | --- | --- | --- | --- | --- | --- | --- | --- | --- | --- | --- | --- |
| **Classification based BMI** **percentiles** | | | | | | | | | | | | |
| Normal weight  (293 boys) | Mean | | 610 | 469.9 | 42.9 | 10.0 | 51.9 | 9494 | 97.3 | 573.6 | 14.3 | 5.0 |
|  | SD | | 150 | 86.3 | 19.2 | 9.2 | 24.1 | 2603 | 1.6 | 61.0 | 6.6 | 2.0 |
|  | Median | | 612 | 479.1 | 40.7 | 8.2 | 48.5 | 9289 | 97.7 | 578.7 | 13.2 | 5.0 |
|  | IQR | | 157 | 78.3 | 21.0 | 8.2 | 28.4 | 2934 | 1.7 | 74.1 | 8.0 | 3.0 |
| Excess weight  (38 boys) | Mean | | 574 | 474.5 | 37.1 | 7.5 | 44.3 | 8454 | 96.3 | 543.6 | 17.1 | 6.0 |
|  | SD | | 116 | 88.5 | 17.6 | 6.6 | 21.8 | 1859 | 2.5 | 68.2 | 6.9 | 2.0 |
|  | Median | | 586 | 480.6 | 37.0 | 6.0 | 43.6 | 8132 | 96.7 | 548.6 | 18.0 | 6.0 |
|  | IQR | | 172 | 91.0 | 19.4 | 5.9 | 29.3 | 2258 | 2.1 | 92.8 | 10.1 | 4.0 |
| ***p*** | | | 0.101 | 0.946 | 0.072 | **0.041** | 0.058 | **0.005** | **0.002** | **0.019** | **0.012** | **0.003** |
| **Classification based BFP percentiles** | | | | | | | | | | | | |
| No excess adiposity  (149 boys) | | Mean | 628 | 463.5 | 45.1 | 11.9 | 55.6 | 9930 | 97.3 | 577.5 | 14.6 | 5.0 |
|  |  | SD | 158 | 99.9 | 19.8 | 11.0 | 24.7 | 2773 | 1.6 | 62.8 | 6.6 | 2.0 |
|  |  | Median | 626 | 482.7 | 42.0 | 9.1 | 52.0 | 9718 | 97.6 | 583.4 | 13.4 | 5.0 |
|  |  | IQR | 168 | 85.1 | 22.9 | 9.1 | 30.7 | 3225 | 1.7 | 66.9 | 8.1 | 3.0 |
| Excess adiposity  (182 boys) | | Mean | 588 | 476.1 | 39.9 | 8.0 | 47.3 | 8920 | 97.1 | 564.1 | 14.6 | 5.0 |
|  |  | SD | 132 | 73.5 | 18.2 | 6.4 | 22.6 | 2256 | 1.8 | 61.7 | 6.8 | 2.0 |
|  |  | Median | 598 | 478.3 | 38.1 | 6.5 | 45.8 | 8853 | 97.5 | 569.7 | 13.6 | 5.0 |
|  |  | IQR | 153 | 73.3 | 20.6 | 7.5 | 26.7 | 2577 | 1.9 | 85.1 | 9.2 | 3.0 |
| ***p*** | | | **0.006** | 0.935 | **0.014** | **<0.001** | **0.003** | **0.001** | 0.487 | 0.069 | 0.958 | 0.555 |

**BFP** – body fat percentage; **BMI** – body mass index; **MVPA** – moderate to vigorous physical activity; **WASO** – wake after sleep onset; significant associations are highlighted in bold. Activity levels were determined based on triaxial 24h-accelerometry vector magnitude, defining 101–2295 cpm as light PA, 2296–4011 cpm as moderate PA, ≥ 4012 cpm as vigorous PA and ≥ 2296 cpm as MVPA^29^. Excess weight was defined as BMI ≥ 85th percentile; normal weight was defined as BMI < 85th percentile^33^. Excess adiposity was defined as BFP ≥ 85th percentile; no excess adiposity was defined as BMI < 85th percentile^37^.

Supplementary table S2. Physical activity and sleep parameters in girls with different body weight and adiposity levels based on the 85th percentile.

| **Variable** | | **Total PA (cpm)** | **Light PA (min/day)** | **Moderate PA (min/day)** | **Vigorous PA (min/day)** | **MVPA (min/day)** | **Steps/ day** | **Sleep efficiency (%)** | **Sleep duration (min)** | **WASO (min)** | **Number of awakenings** |
| --- | --- | --- | --- | --- | --- | --- | --- | --- | --- | --- | --- |
| **Classification based BMI** **percentiles** | | | | | | | | | | | |
| Normal weight  (307 girls) | Mean | 582 | 464.9 | 35.1 | 9.7 | 44.3 | 9010 | 97.5 | 580.9 | 14.0 | 5.0 |
|  | SD | 130 | 67.3 | 13.7 | 8.6 | 19.3 | 2011 | 1.2 | 67.2 | 5.9 | 2.0 |
|  | Median | 574 | 470.3 | 34.1 | 7.3 | 41.1 | 8751 | 97.7 | 588.0 | 13.5 | 5.0 |
|  | IQR | 166 | 62.9 | 18.8 | 7.7 | 25.2 | 2653 | 1.6 | 74.0 | 8.1 | 3.0 |
| Excess weight  (38 girls) | Mean | 583 | 458.5 | 34.8 | 9.1 | 43.8 | 8906 | 97.4 | 550.3 | 13.7 | 5.0 |
|  | SD | 143 | 60.5 | 20.6 | 9.0 | 28.4 | 2534 | 1.4 | 83.6 | 6.5 | 2.0 |
|  | Median | 576 | 473.9 | 30.4 | 7.3 | 37.7 | 8392 | 97.5 | 554.3 | 12.5 | 4.0 |
|  | IQR | 187 | 65.1 | 22.0 | 8.2 | 25.0 | 3482 | 2.2 | 95.8 | 9.8 | 3.0 |
| ***p*** | | 0.890 | 0.563 | 0.547 | 0.425 | 0.486 | 0.719 | 0.741 | **0.017** | 0.682 | 0.328 |
| **Classification based BFP percentiles** | | | | | | | | | | | |
| No excess adiposity (234 girls) | Mean | 591 | 465.1 | 35.4 | 10.4 | 45.3 | 9150 | 97.5 | 582.1 | 13.8 | 5.0 |
|  | SD | 137 | 70.6 | 14.4 | 9.3 | 20.5 | 2022 | 1.2 | 66.6 | 6.1 | 2.0 |
|  | Median | 584 | 469.5 | 33.8 | 7.6 | 42.2 | 8913 | 97.7 | 589.1 | 13.3 | 5.0 |
|  | IQR | 174 | 65.0 | 21.6 | 8.7 | 27.2 | 2579 | 1.6 | 72.3 | 8.5 | 3.0 |
| Excess adiposity (111 girls) | Mean | 564 | 462.3 | 34.3 | 8.1 | 41.9 | 8679 | 97.3 | 567.9 | 14.3 | 5.0 |
|  | SD | 118 | 57.4 | 15.2 | 6.8 | 20.3 | 2144 | 1.3 | 75.3 | 5.8 | 2.0 |
|  | Median | 553 | 473.7 | 33.6 | 6.7 | 38.4 | 8400 | 97.5 | 577.4 | 13.8 | 5.0 |
|  | IQR | 148 | 62.5 | 16.9 | 6.3 | 19.4 | 2981 | 1.8 | 74.2 | 8.6 | 3.0 |
| ***p*** | | 0.065 | 0.608 | 0.463 | **0.025** | 0.193 | **0.029** | 0.262 | 0.085 | 0.328 | 0.348 |

**BFP** – body fat percentage; **BMI** – body mass index; **MVPA** – moderate to vigorous physical activity; **WASO** – wake after sleep onset; significant associations are highlighted in bold. Activity levels were determined based on triaxial 24h-accelerometry vector magnitude, defining 101–2295 cpm as light PA, 2296–4011 cpm as moderate PA, ≥ 4012 cpm as vigorous PA and ≥ 2296 cpm as MVPA^29^. Excess weight was defined as BMI ≥ 85th percentile; normal weight was defined as BMI < 85th percentile^33^. Excess adiposity was defined as BFP ≥ 85th percentile; no excess adiposity was defined as BMI < 85th percentile^37^.
